# Dementia Nursing Competency Scale at Acute Hospitals Version 2: Development and Psychometric Evaluation

**DOI:** 10.1093/geroni/igaa057.445

**Published:** 2020-12-16

**Authors:** Chieko Greiner, Shoko Urashima, Hirochika Ryuno, Yuko Yamaguchi, Atsuko Fukuda

**Affiliations:** 1 KOBE UNIVERSITY, Kobe, Japan; 2 Osaka Saiseikai Ibaraki Hospital, Osaka, Japan; 3 Kobe University, Kobe, Japan; 4 Kobe University, Kobe, United States

## Abstract

Under an increasing number of people with dementia worldwide, the number of patients with dementia in acute settings is also growing. For patients with dementia to receive treatment with peace of mind, nurses should have competency in caring for patients with dementia. Thus, the aims of this study were to develop the Dementia Nursing Competency Scale in Acute Hospitals version 2 (DNCS-AH-v2) and to evaluate its psychometric properties. The draft was distributed to a convenience sample of 3000 nurses at 300 acute hospitals. After confirming the ceiling effect and the floor effect, reliability and validity were verified using Cronbach’s alpha, I-T correlation, test-retest reliability, G-P analysis, and exploratory and confirmatory factor analyses. This study was approved by the Institutional Review Board of Kobe University. Of a total of 3000 nurses, 878 (29.3%) responded to the questionnaire and 773 (25.8%) valid responses were analyzed. Mean age and mean working experience of the participants were 37.4 ± 9.3 and 14.0 ± 8.7 years, respectively. The 27-item DNCS-AH-v2 was developed with five factors scored on a six-point Likert scale. Cronbach’s alpha was .925, I-T correlation was between .46 and .68 (p<.01), ICC coefficient (1, 1) was .76 (p<.001), and significant differences were confirmed for G-P analysis (p<.001). For the confirmatory factor analysis, fit indices of CFI =.868, GFI =.868, and RMSEA =.065 were obtained. High reliability and moderate validity were confirmed for the DNCS-AH-v2. The developed DNCS-AH-v2 could be used to evaluate dementia nursing competency in acute hospitals.

